# Supplementary material for: Lem2 and Lnp1 maintain the membrane boundary between the nuclear envelope and endoplasmic reticulum
Source: Commun Biol. 2020 Jun 1;3:276. doi: 10.1038/s42003-020-0999-9 (PMC7264229; doi:10.1038/s42003-020-0999-9)
Supplement: Supplementary file 1 — Description of Additional Supplementary Files [file 42003_2020_999_MOESM1_ESM.pdf]

Description of Additional Supplementary Files

File Name: **Supplementary Movie 1**
Description: **Behavior of nuclear protein in *lem2*Δ*lnp1*Δ cells**
Time-lapse images of *lem2*Δ*lnp1*Δ cells expressing GFP-GST-NLS (green) and Ish1-mCherry and Atb2-mCherrry (magenta) were obtained every 5 min. Optical section images at 1-μm focal interval were obtained at each timepoint and projected using the maximum intensity method. Projected images after denoising and deconvolution are presented. Scale bar represents 10 μm.

File Name: **Supplementary Movie 2**
Description: **Behavior of nuclear protein in WT cells**
Time-lapse images of WT cells expressing GFP-GST-NLS (green) and Ish1-mCherry and Atb2-mCherrry (magenta) were obtained as described in Movie 1. Scale bar represents 10 μm.

File Name: **Supplementary Movie 3**
Description: **Behavior of nuclear protein in *lem2*Δ cells**
Time-lapse images of *lem2*Δ cells expressing GFP-GST-NLS (green) and Ish1-mCherry and Atb2-mCherrry (magenta) were obtained as described in Movie 1. Scale bar represents 10 μm.

File Name: **Supplementary Movie 4**
Description: **Behavior of nuclear protein in *lnp1*Δ cells**
Time-lapse images of *lnp1*Δ cells expressing GFP-GST-NLS (green) and Ish1-mCherry and Atb2-mCherrry (magenta) were obtained as described in Movie 1. Scale bar represents 10 μm.

File Name: **Supplementary Movie 5**
Description: **Behavior of nuclear protein in *lem2*Δ*lnp1*Δ cells overexpressing Apq12**
Time-lapse images of Apq12-overexpressing *lem2*Δ*lnp1*Δ cells expressing GFP-GST-NLS (green) and Ish1-mCherry and Atb2-mCherrry (magenta) were obtained as described in Movie 1. Scale bar represents 10 μm.

File Name: **Supplementary Movie 6**
Description: **Behavior of nuclear protein in *cmp7*Δ cells**
Time-lapse images of *cmp7*Δ cells expressing GFP-GST-NLS (green) and Ish1-mCherry and Atb2-mCherrry (magenta) were obtained as described in Movie 1. Scale bar represents 10 μm.

File Name: **Supplementary Data 1**
Description: ***S. pombe*** **strains used in this study**
