# Supplementary material for: Lem2 and Lnp1 maintain the membrane boundary between the nuclear envelope and endoplasmic reticulum
Source: Commun Biol. 2020 Jun 1;3:276. doi: 10.1038/s42003-020-0999-9 (PMC7264229; doi:10.1038/s42003-020-0999-9)
Supplement: Supplementary file 2 — Supplementary Information [file 42003_2020_999_MOESM2_ESM.pdf]

**Supplementary Information**

# Lem2 and Lnp1 maintain the membrane boundary between the nuclear envelope and endoplasmic reticulum

**Supplementary Figures**

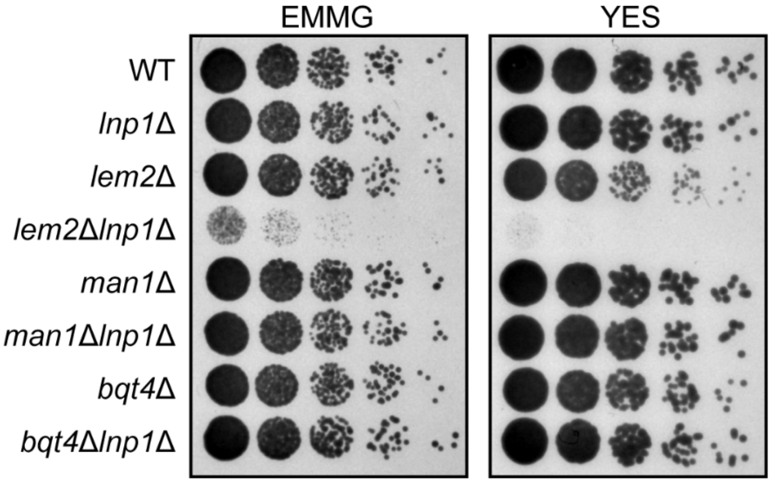


**Supplementary Figure 1.** ***lem2***$^{+}$**, but not** ***man1***$^{+}$ **and** ***bqt4***$^{+}$**, genetically interacts with** ***lnp1***$^{+}$

Spot assay results of wild type (WT), single- and double-deletion mutants. Five-fold serially diluted cells were spotted on minimum (EMMG) and rich (YES) medium plates and incubated for 3 and 2 days, respectively.

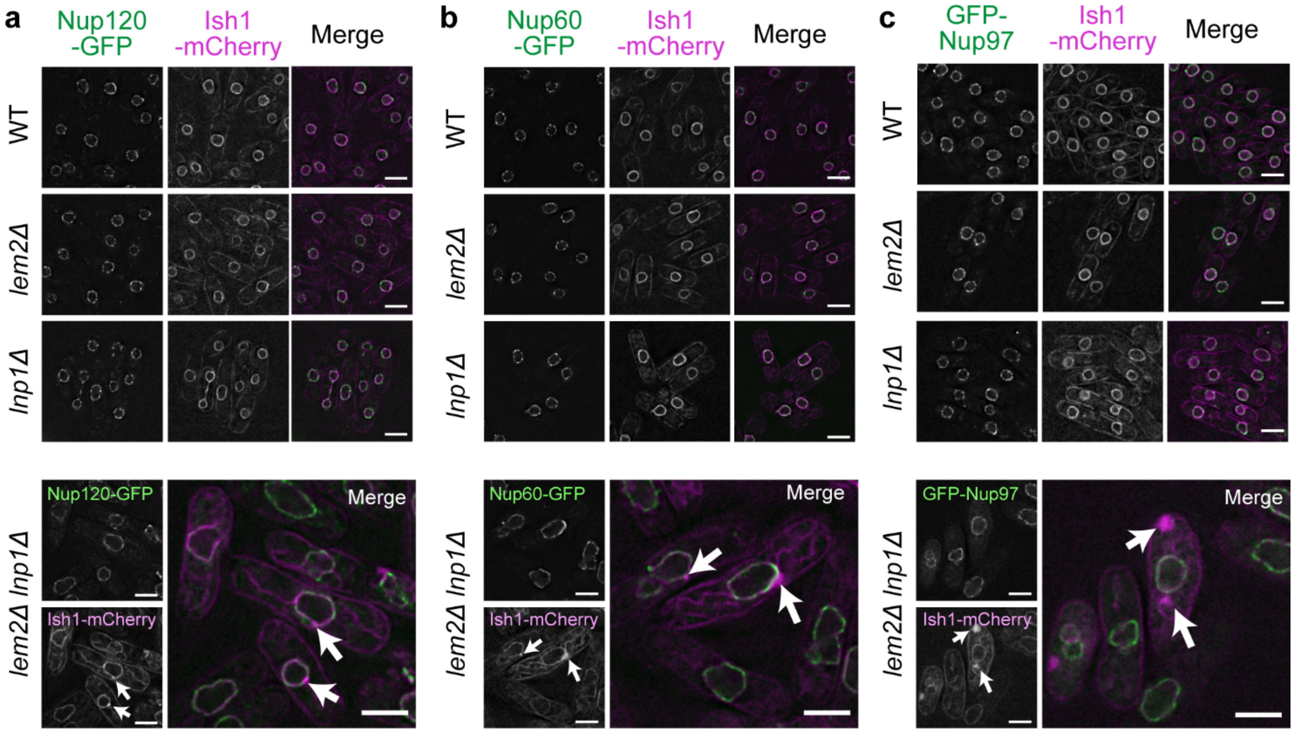


**Supplementary Figure 2. Double deletion of *lem2*$^{+}$ and *lnp1*$^{+}$ has no effect on the localization of NPC components**

One of the NPC components fused with GFP was observed in WT, *lem2*Δ*, lnp1*Δ, and *lem2*Δ*lnp1*Δ cells expressing Ish1-mCherry. **a**, Nup120-GFP. **b**, Nup60-GFP. **c**, GFP-Nup97, Nup120, Nup60, and Nup97 were present at the cytoplasmic side of the outer ring, nuclear basket, and inner ring, respectively (Asakawa *et al.*, 2019) [1]. Arrows indicate positions of the abnormal nuclear membrane. Scale bar represents 5 µm.

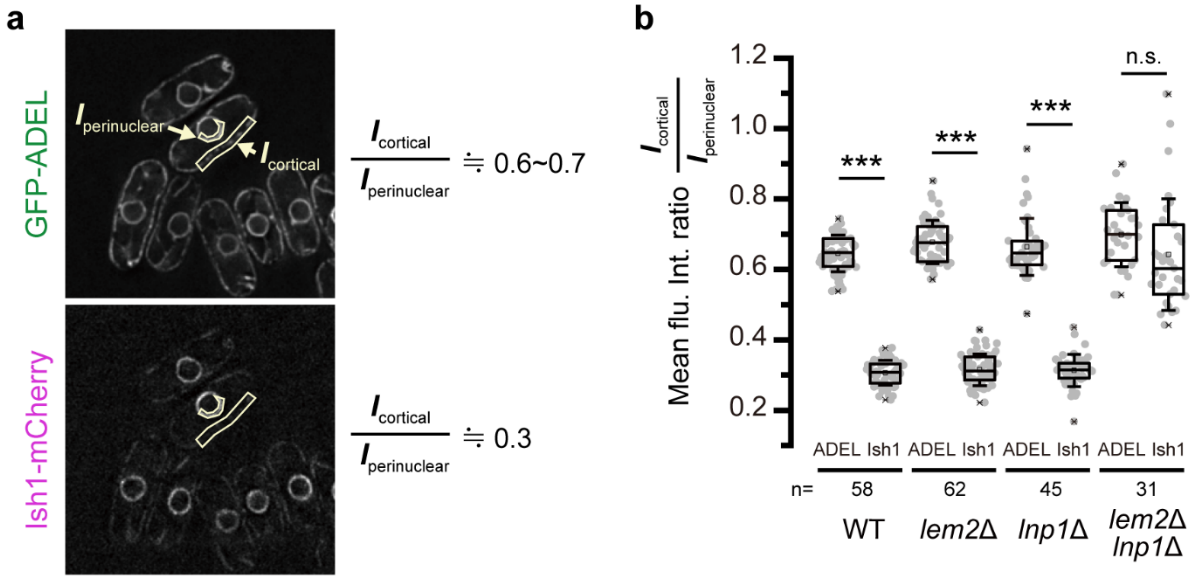


**Supplementary Figure 3. Quantification of NE and ER localization of Ish1**

**a**. Quantification procedure: Perinuclear and cortical ER regions were manually marked in the GFP-ADEL image (indicated by yellow). Fluorescence intensities in these regions ($I_{perinuclear}$ and $I_{cortical}$) were measured, and the ratio ($I_{cortical}/I_{perinuclear}$) was calculated. Fluorescence intensities of Ish1-mCherry at the exact same positions were also measured, and the ratio was also calculated. Typical $I_{cortical}/I_{perinuclear}$ values of GFP-ADEL and Ish1-mCherry in WT cells were 0.6-0.7 and 0.3, respectively. **b**. The ratios ($I_{cortical}/I_{perinuclear}$) were plotted for GFP-ADEL (ADEL) and Ish1-mCherry (Ish1) in WT, *lem2Δ, lnp1Δ*, and *lem2Δlnp1Δ* cells. Gray dots represent individual values. In a box-and-whisker plot, horizontal lines in the box indicate the upper quartile, median, and lower quartile, from top to bottom, respectively; the whisker indicates standard deviation. n indicates the total cell number counted. P values from an unpaired two-tailed Student's *t*-test. ***: $p < 0.001$ (WT: $p=5.5\times 10^{-70}$, *lem2*Δ: $p=2.8\times 10^{-65}$, *lnp1*Δ: $p=4.7\times 10^{-42}$), n.s.: no significance (*lem2*Δ*lnp1*Δ: $p=0.091$).

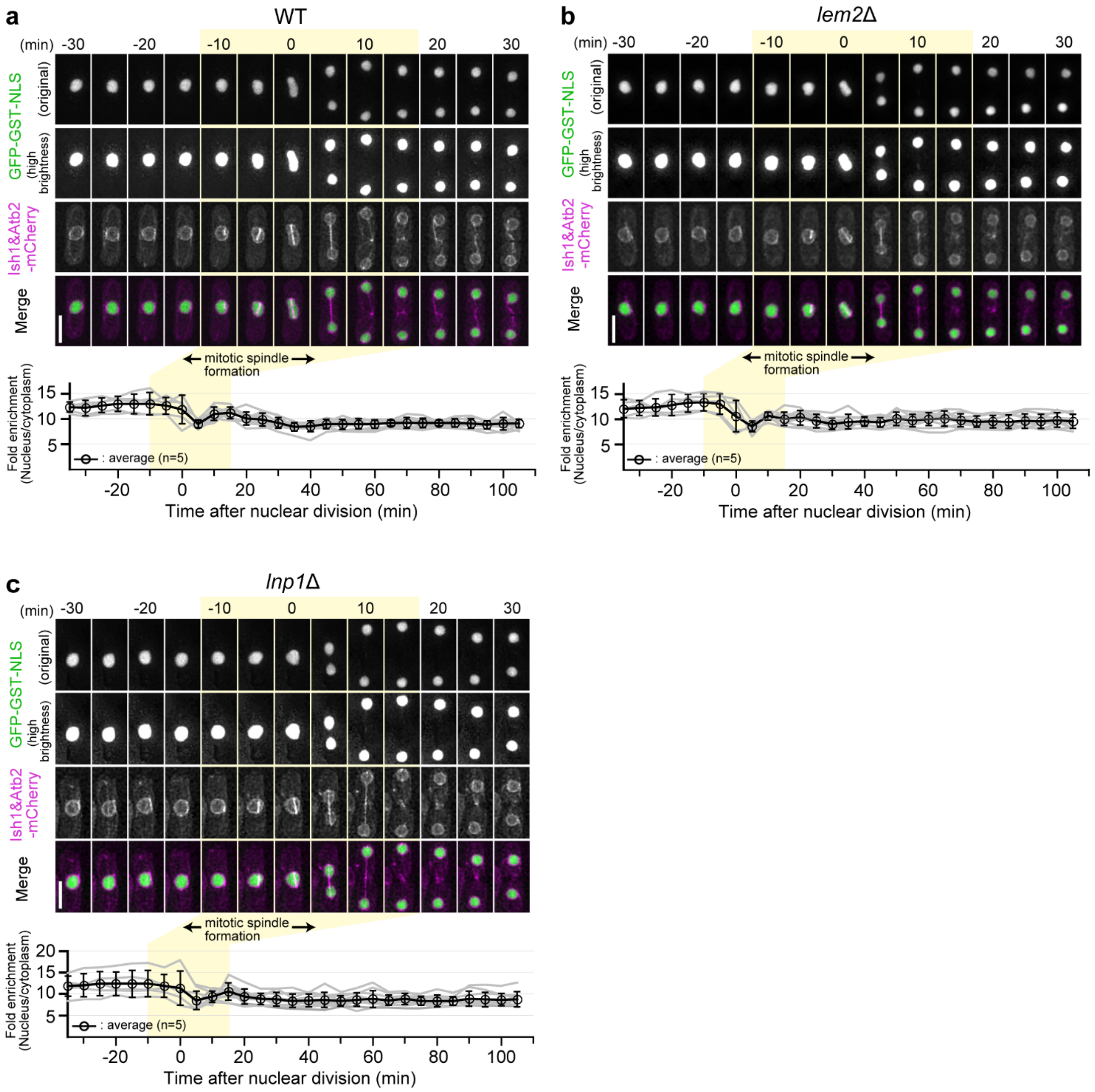


**Supplementary Figure 4. No obvious nuclear protein leakage in WT, *lem2*Δ, and *lnp1*Δ cells**

The WT (**a**), *lem2*Δ (**b**), and *lnp1*Δ (**c**) cells expressing GFP-GST-NLS, Ish1-mCherry, and Atb2-mCherry were observed in a living state, as described in Fig. 2**f**. Fold enrichment of GFP-GST-NLS was quantified and shown at the bottom. Black line represents mean ± standard deviation of five independent quantifications. Gray lines represent individual quantifications. Scale bar represents 5 µm.

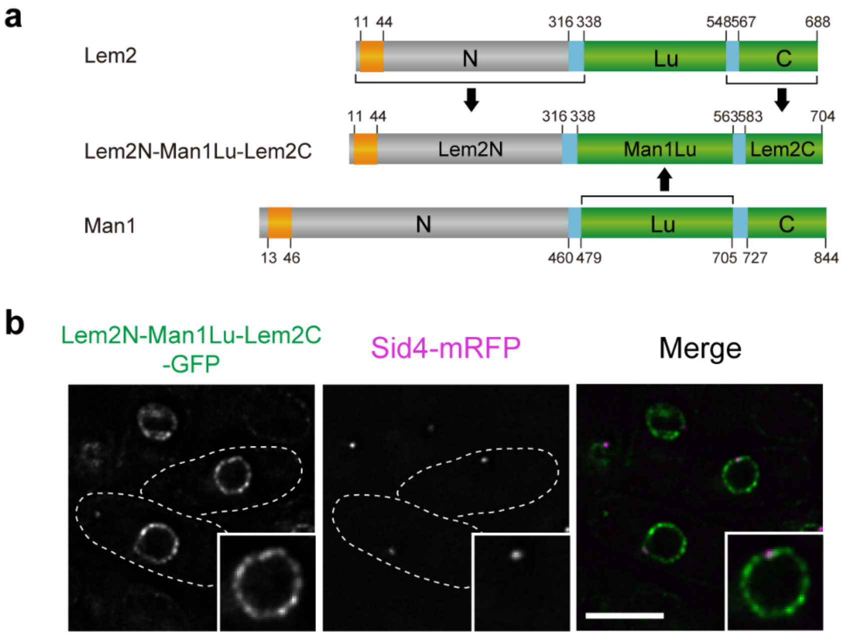


**Supplementary Figure 5. Lem2N-Man1Lu-Lem2C localized at the NE**

**a**. Schematic illustration of Lem2 fragments. The luminal region of Lem2 (338-548 a.a., top) is substituted to the corresponding region of Man1 (479-705 a.a., bottom).

**b**. Lem2N-Man1Lu-Lem2C localization. GFP-tagged Lem2N-Man1Lu-Lem2C (green) was expressed with Sid4-mRFP (SPB marker, magenta) in *S. pombe* cells. Insets represent enlarged images of the nucleus. The dashed lines indicate the outline of the cells. Scale bar represents 5 μm.

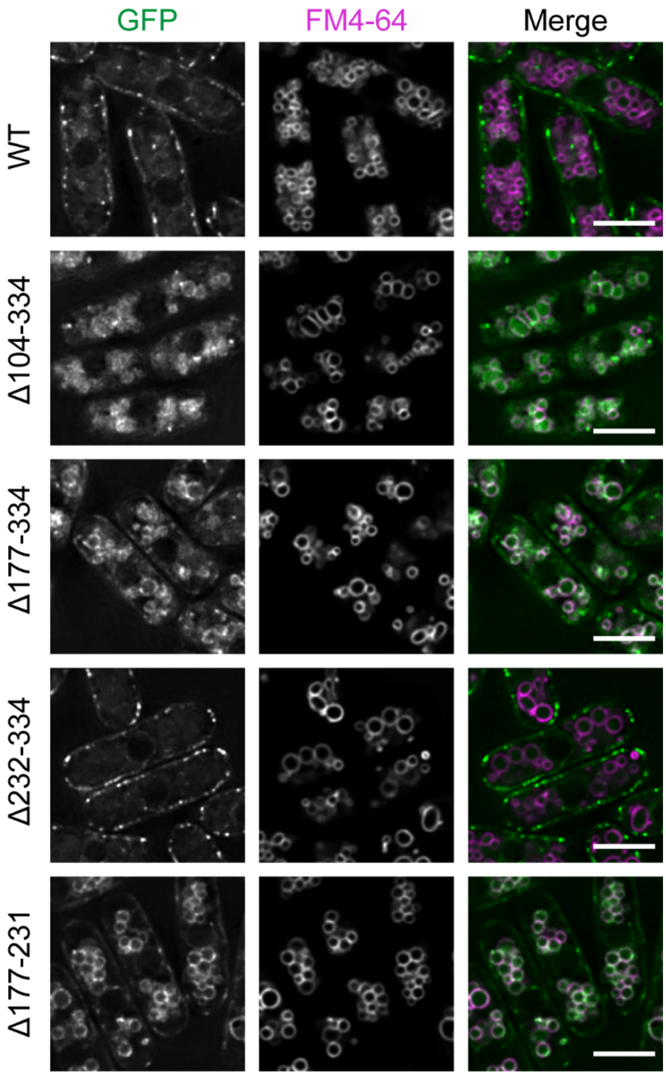


**Supplementary Figure 6. Lunapark domain-deleted mutants of Lnp1 localize at the vacuole membrane**

GFP-tagged WT and deletion mutants of Lnp1 (green) were expressed in the wild type cells and stained with FM4-64 (magenta). Deconvolved single-section images are shown. Scale bar represents 5 μm.

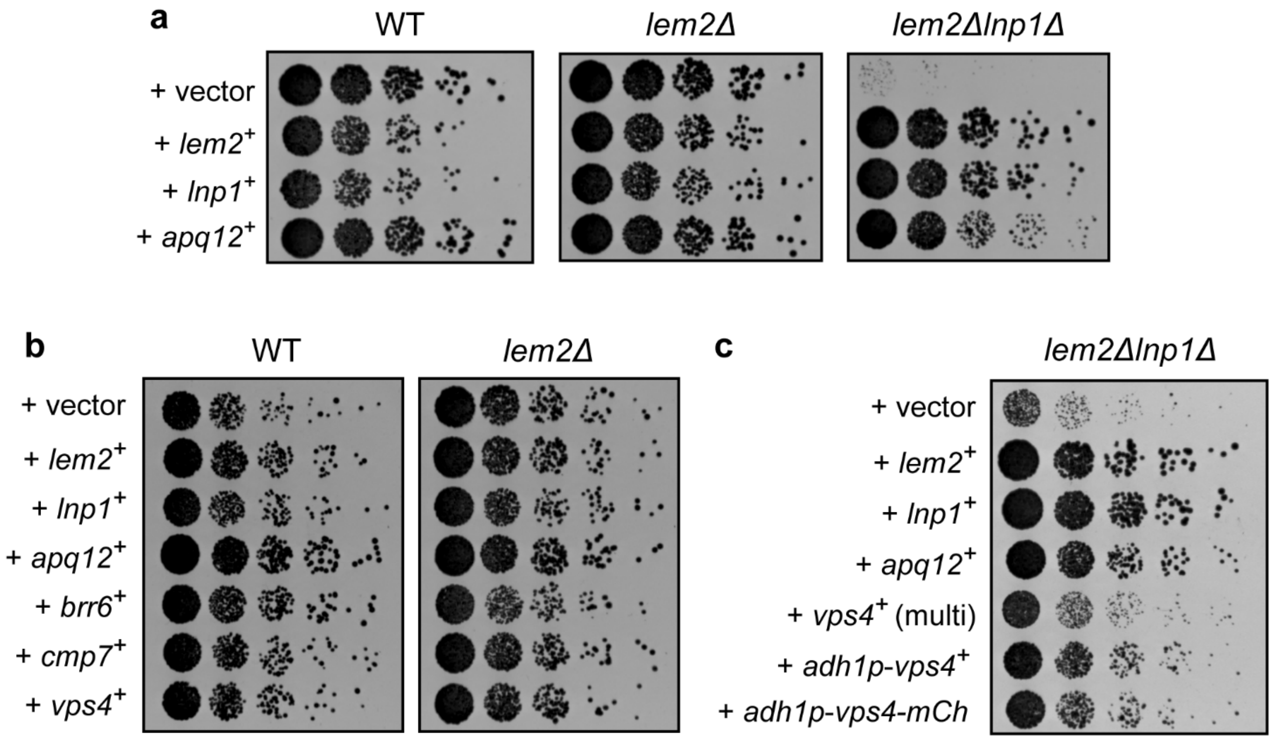


**Supplementary Figure 7. Effect of gene expression on cell growth**

**a**. Effect of Apq12 overexpression. The genes indicated on the left were expressed by the multicopy plasmid in WT (left), *lem2*Δ (middle), and *lem2*Δ*lnp1*Δ (right) cells. The vector plasmid was expressed as a control. Five-fold serially diluted cells indicated on the left were spotted on EMMG plates and incubated for 3 days.

**b**. Effect of Brr6, Cmp7, and Vps4 overexpression. The genes indicated on the left were expressed by the multi-copy plasmid in WT (left) and *lem2*Δ (right) cells. A spot assay was performed as described in (**a**).

**c**. Effect of Vps4 overexpression in *lem2*Δ*lnp1*Δ cells. Vps4 was overexpressed from a multi-copy plasmid (*vps*4$^{+}$-multi) or the *leu1*$^{+}$ genomic locus driven by the constitutive *adh1* promoter with (*adh1p-vps4-mCh*) or without mCherry tagging (*adh1p*-*vps4*$^{+}$) in *lem2*Δ*lnp1*Δ cells. A spot assay was performed as described in (**a**).

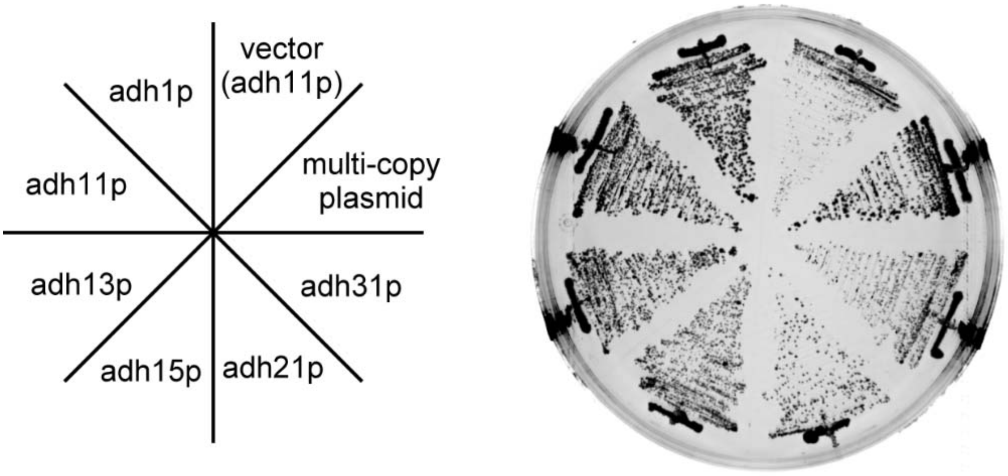


**Supplementary Figure 8. Choice of promoters for Apq12 expression to restore the growth defect of *lem2*Δ*lnp1*Δ cells**

The *apq12*$^{+}$ gene was fused with different strengths of constitutive promoters (*adh1p, adh11p, adh13p, adh15p, adh21p*, and *adh31p* in decreasing order of strength) and integrated into *lem2*Δ*lnp1*Δ cells at the *leu1*$^{+}$ site in the genome. The left panel represents the promoters used in the right panel. The right panel shows cell growth on EMMG plates incubated for 4 days. Growth of *lem2*Δ*lnp1*Δ cells expressing Apq12 under *adh1p* and *adh11p* almost fully restored to a level of Apq12 expressed on a multi-copy plasmid.

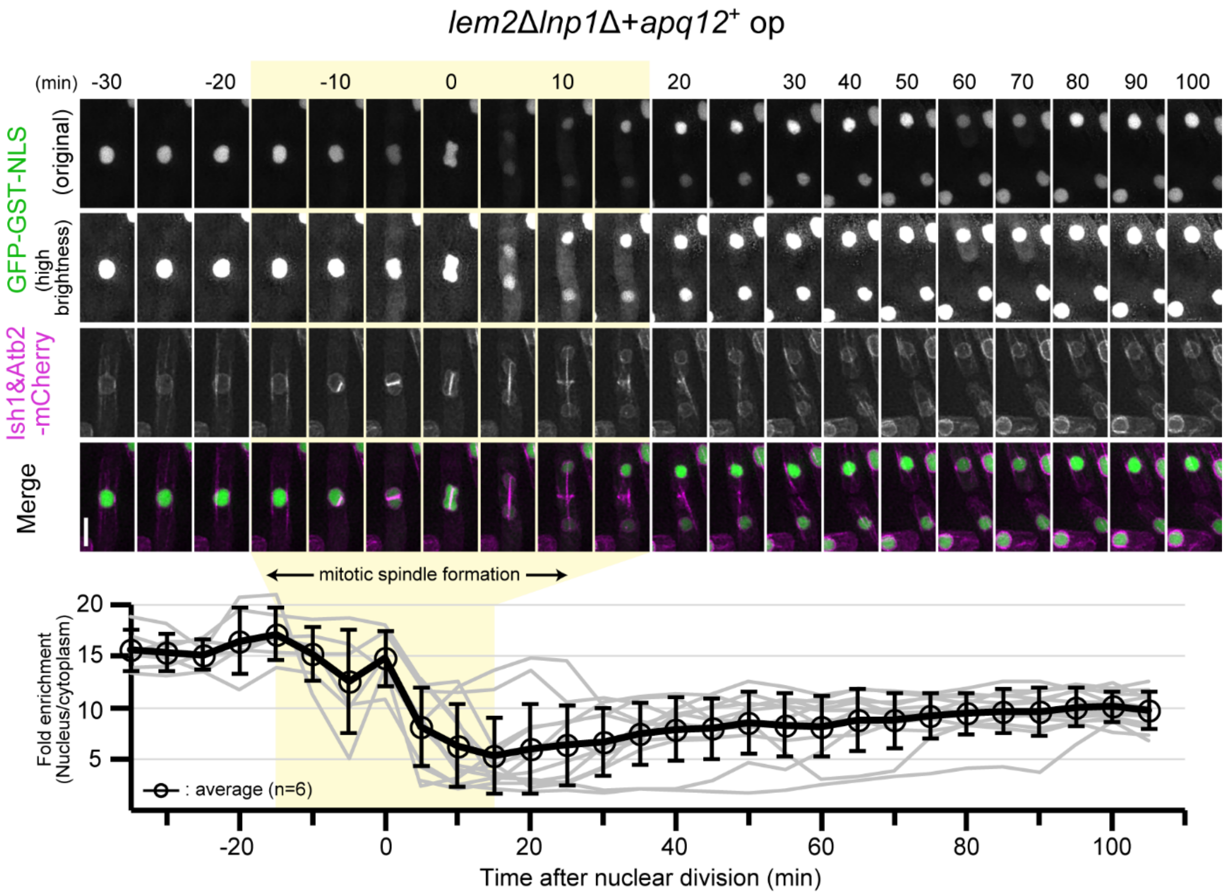


**Supplementary Figure 9. Apq12 overexpression does not restore mitotic nuclear protein leakage in *lem2*Δ*lnp1*Δ cells**

GFP-GST-NLS, Ish1-mCherry, and Atb2-mCherry were expressed in Apq12-overexpressing *lem2*Δ*lnp1*Δ cells and observed in the living state as described in Fig. 2**f**. Fold enrichment of GFP-GST-NLS was quantified and plotted at the bottom. Open circles and error bars represent mean ± standard deviation of six independent quantifications. Gray lines represent six individual quantifications. Scale bar represents 5 μm.

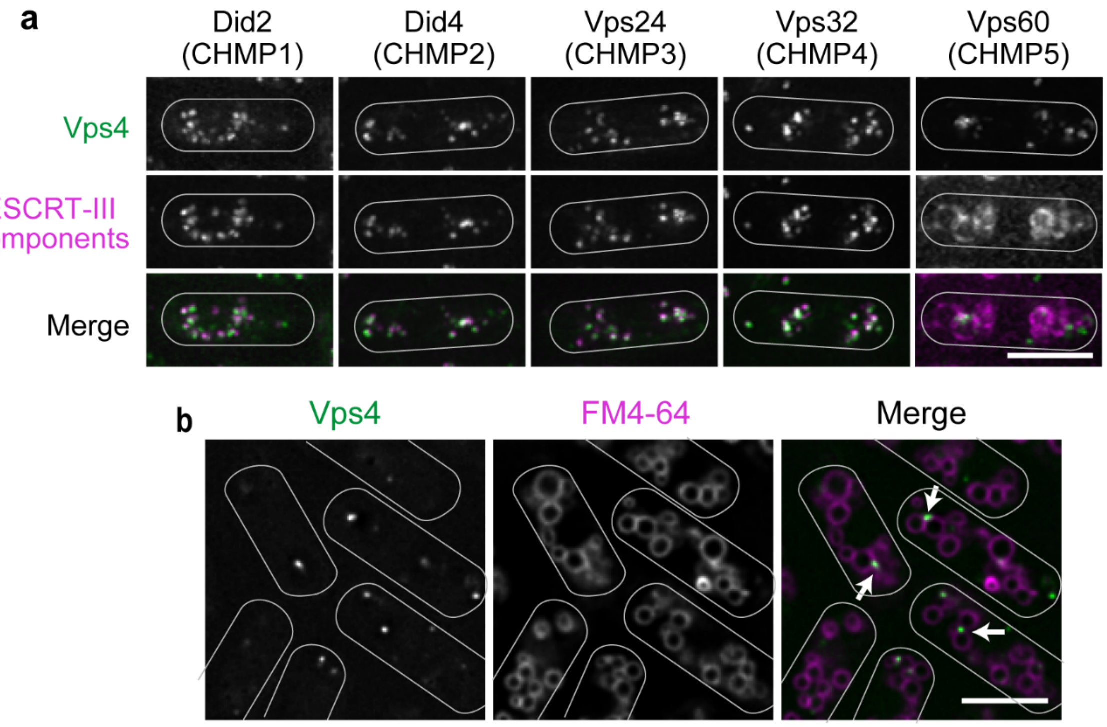


**Supplementary Figure 10. Vps4 colocalizes with the ESCRT-III complex in WT cells**

**a**. *S. pombe* cells were co-expressed with Vps4-GFP (green) and mCherry-tagged ESCRT-III components (Did2, Did4, Vps24, Vps32 or Vps60, magenta) indicated on the top and observed by fluorescence microscopy. The names in parentheses indicate those in human homologs. Projected images after deconvolution are shown. The cell shape is outlined by white line. Scale bar represents 5 μm.

**b**. Cells expressing Vps4-GFP (green) were stained with FM4-64 (magenta). Arrows indicate Vps4 dots adjacent to the vacuole membrane. Projected images after deconvolution are shown. The cell shape is outlined by white line. Scale bar represents 5 μm.

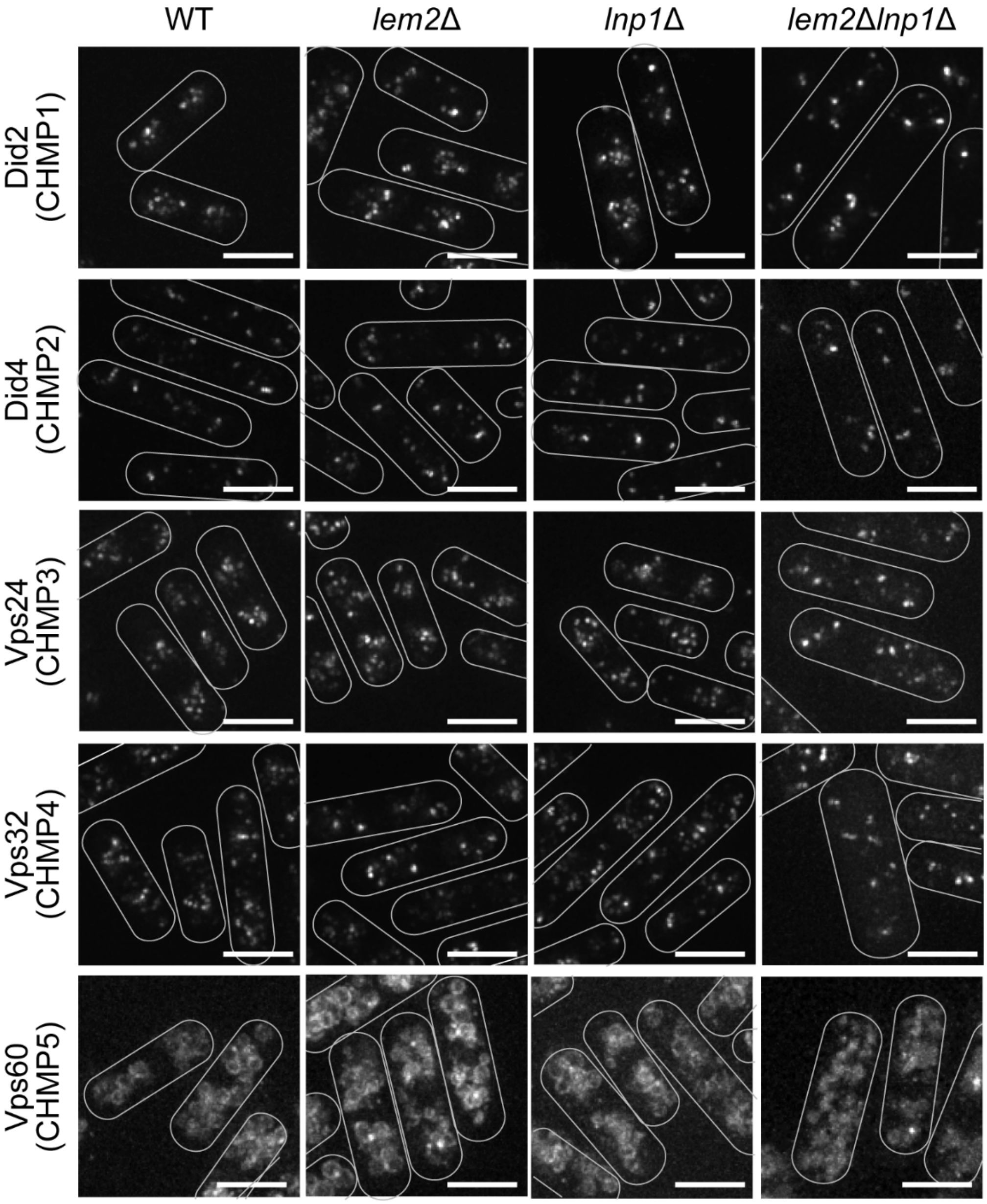


**Supplementary Figure 11. Double deletion of *lem2*[+] and *lnp1*[+] genes has no effect on the localization of ESCRT-III components**

mCherry-tagged Did2, Did4, Vps24, Vps32, or Vps60 were expressed in WT, *lem2*Δ, *lnp1*Δ, and *lem2*Δ*lnp1*Δ cells. The cells were observed by fluorescence microscopy. Deconvolved single-section images are shown. Scale bar represents 5 μm.

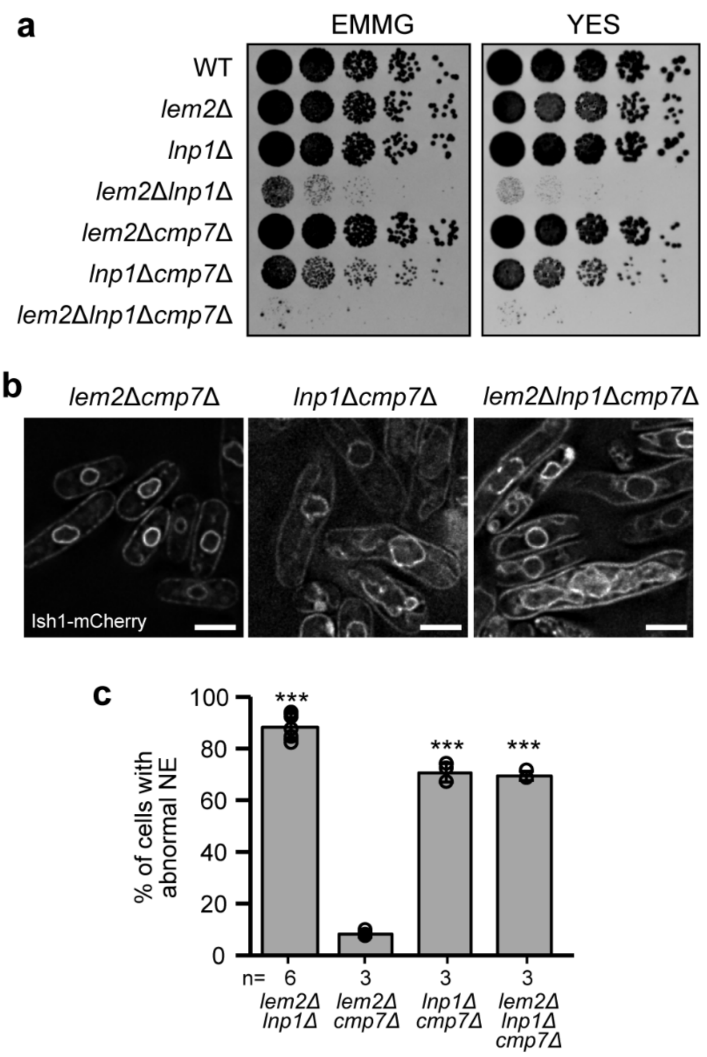


**Supplementary Figure 12. Deletion of *cmp7*⁺ does not restore growth and membrane defects in *lem2*Δ*lnp1*Δ cells**

**a.** Growth of cells indicated on the left. Five-fold serially diluted cells were spotted on the EMMG or YES plate, and growth of these cells was observed after 3 days.

**b.** The indicated mutant cells were labeled with Ish1-mCherry to observe the NE. Scale bar represents 5 μm.

**c**. Percentages of cells with abnormal NE are plotted as mean ± standard deviation. *n* indicates the number of experiments. Open circles represent the percentage of individual experiments. P values from Tukey's test. **: $p < 0.01$, ***: $p < 0.001$

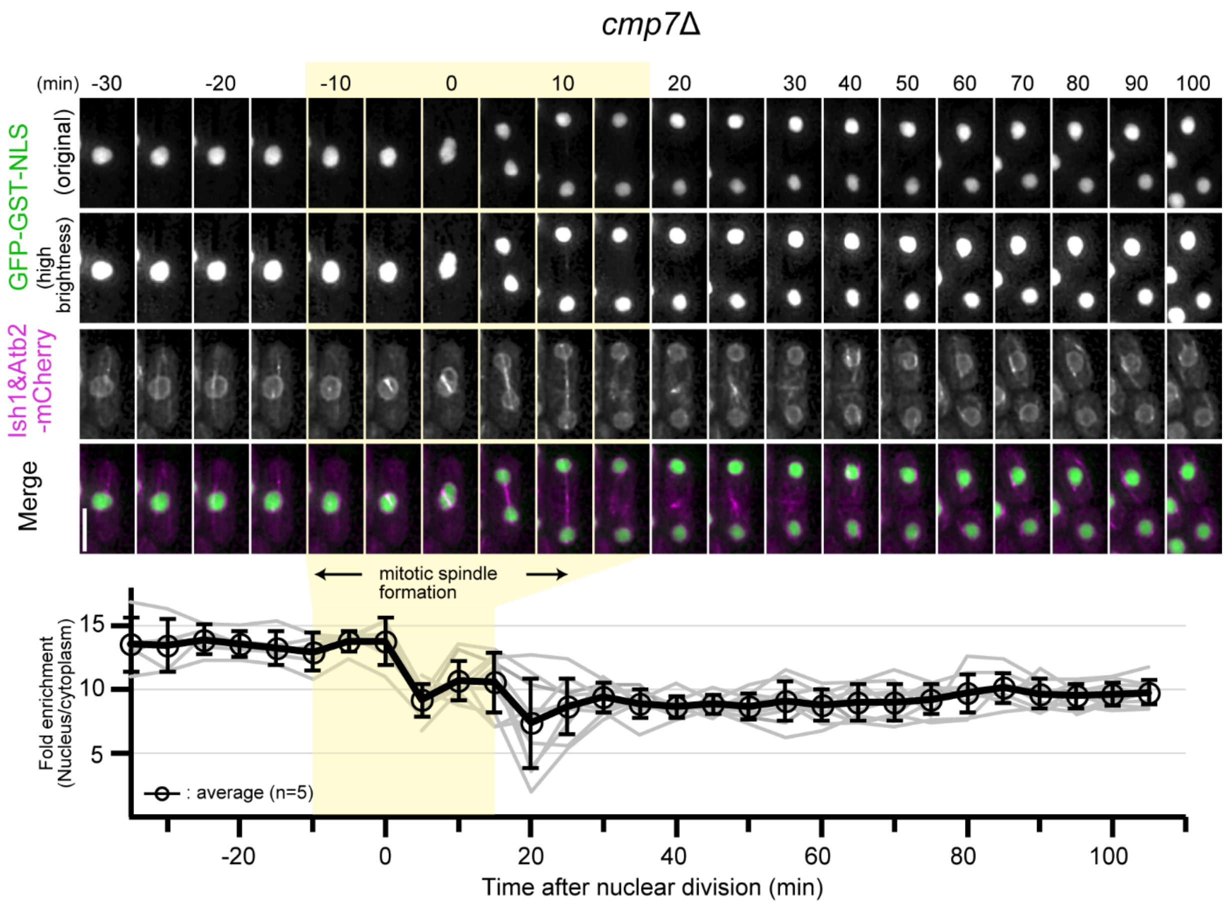


**Supplementary Figure 13. Deletion of the *cmp7*$^{+}$ gene does not show nuclear protein leakage during interphase**

The *cmp7*Δ cells expressing GFP-GST-NLS, Ish1-mCherry, and Atb2-mCherry were observed in a living state, as described in Fig. 2**f**. Fold enrichment of GFP-GST-NLS was quantified and shown at the bottom. Black line represents mean ± standard deviation of five independent quantifications. Gray lines represent individual quantifications. Scale bar represents 5 µm.

Figure 6b

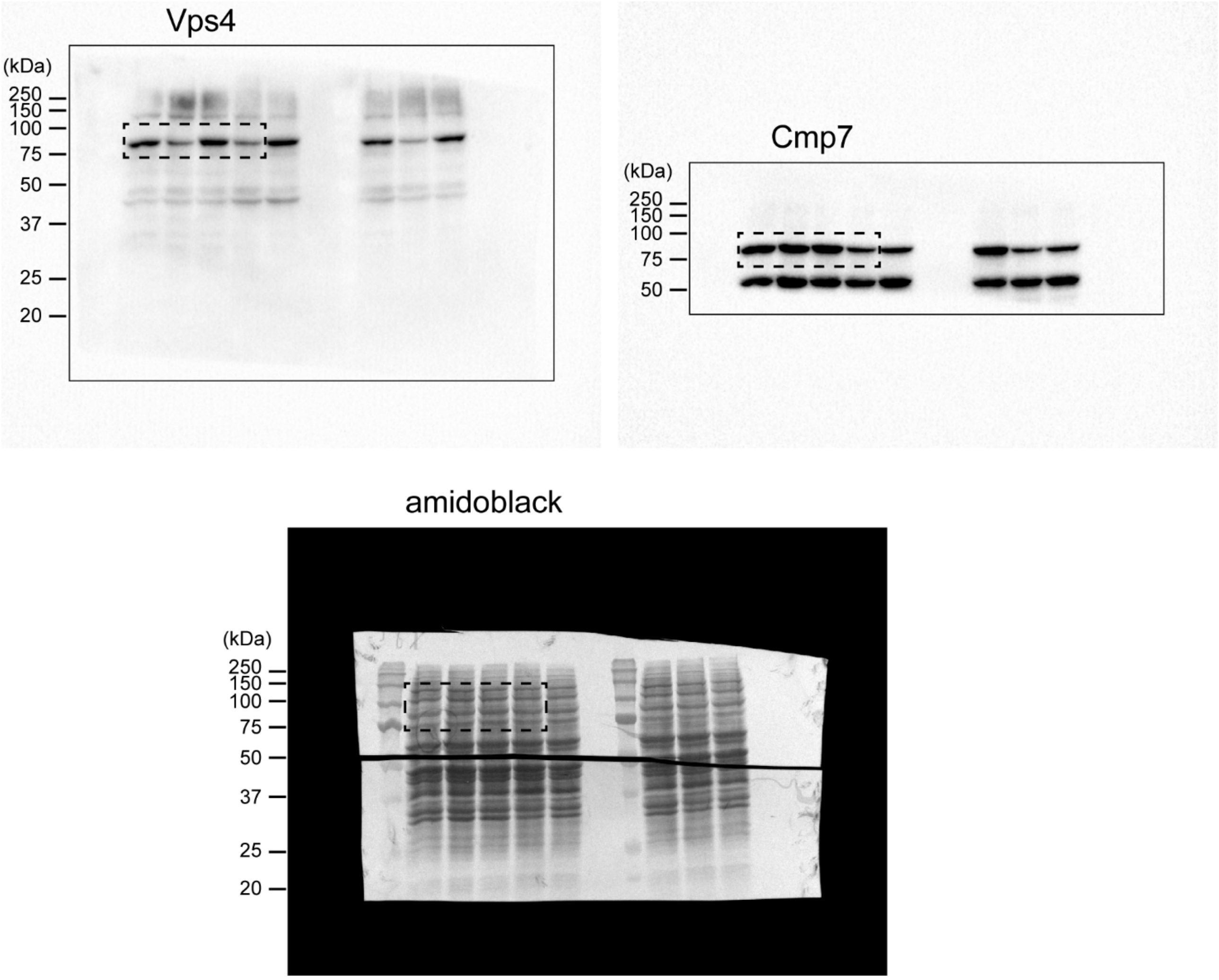


**Supplementary Figure 14. Unprocessed blots**

The continuous and dashed boxes indicate outline of membrane and pictures shown in Figure 6b, respectively.

Figure 8d: *lem2Δ*        Figure 8d: *lem2Δ lnp1Δ*

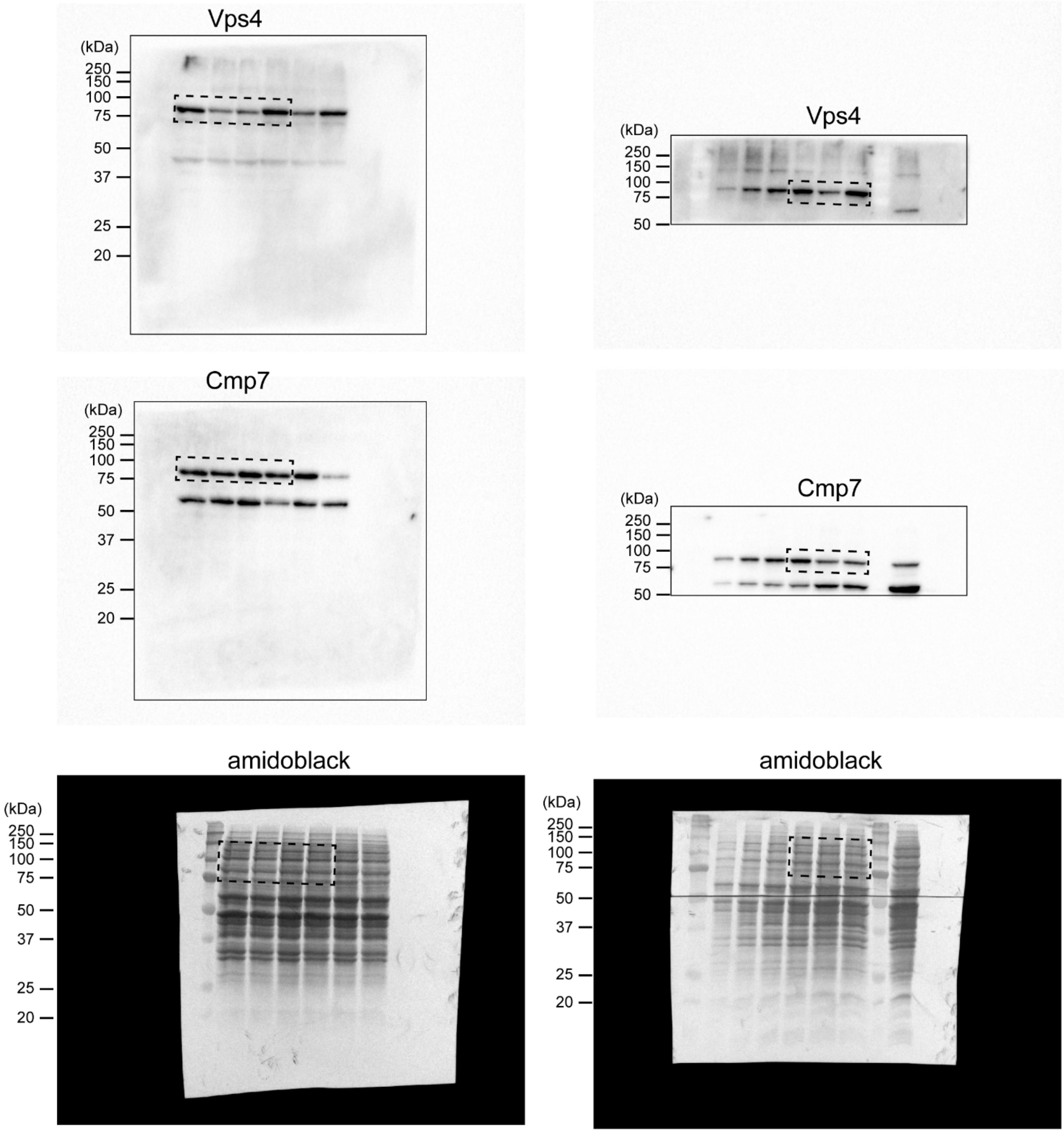


**Supplementary Figure 14 (continued). Unprocessed blots**

The continuous and dashed boxes indicate outline of membrane and pictures shown in Figure 8d, respectively.

## Supplementary References


1. Asakawa, H. *et al.* Asymmetrical localization of Nup107-160 subcomplex components within the nuclear pore complex in fission yeast. *PLoS Genet* **15**, e1008061 (2019).
